# Narrowband Organic/Inorganic Hybrid Afterglow Materials

**DOI:** 10.3390/molecules29102343

**Published:** 2024-05-16

**Authors:** Wen Xia, Xun Li, Junbo Li, Qianqian Yan, Guangming Wang, Xixi Piao, Kaka Zhang

**Affiliations:** 1 College of Chemistry and Materials Science, Sichuan Normal University, Chengdu 610068, China; xiawen@sioc.ac.cn (W.X.); lijunbo@sioc.ac.cn (J.L.); 2 State Key Laboratory of Organometallic Chemistry, Key Laboratory of Synthetic and Self-Assembly Chemistry for Organic Functional Molecules, Shanghai Institute of Organic Chemistry, University of Chinese Academy of Sciences, Chinese Academy of Sciences, 345 Lingling Road, Shanghai 200032, China; lixun@sioc.ac.cn (X.L.); yanqianqian@sioc.ac.cn (Q.Y.); gmw@sioc.ac.cn (G.W.)

**Keywords:** narrowband, afterglow materials, quantum dots, room-temperature phosphorescence

## Abstract

Narrowband afterglow materials display interesting functions in high-quality anti-counterfeiting and multiplexed bioimaging. However, there is still a limited exploration of these afterglow materials, especially for those with a full width at half maxima (FWHM) around 30 nm. Here, we report the fabrication of narrowband organic/inorganic hybrid afterglow materials via energy transfer technology. Coronene (Cor) with a long phosphorescence feature and broad phosphorescence band is selected as the donor for energy transfer, and inorganic quantum dots (QDs) of CdSe/ZnS with a narrowband emission are used as acceptors. Upon doping into the organic matrix, the resultant three-component materials exhibit a narrowband afterglow with an afterglow lifetime of approximately 3.4 s and an FWHM of 31 nm. The afterglow wavelength of the afterglow materials can be controlled by the QDs. This work based on organic/inorganic hybrids provides a facile approach for developing multicolor and narrowband afterglow materials, as well as opens a new way for expanding the features of organic afterglow for multifunctional applications. It is expected to rely on narrowband afterglow emitters to solve the “spectrum congestion” problem of high-density information storage in optical anti-counterfeiting and information encryption.

## 1. Introduction

Long-lived room-temperature phosphorescence (RTP) and organic afterglow materials have attracted increasing attention for their promising applications in anti-counterfeiting, oxygen sensing, organic light-emitting diodes, and biological imaging [1,2,3,4,5,6,7,8,9,10,11]. Due to their high color purity and small FWHM, narrowband afterglow materials can be effectively utilized in high-quality anti-counterfeiting and controllable bio-optical imaging [12,13,14,15,16]. Chen and Huang leveraged the copolymer technology to integrate multi-resonance fluorescence chromophores into polyacrylamide (PAM), achieving an impressive minimum FWHM of 38 nm and ultralong lifetimes of 1.64 s under ambient conditions [17]. An and Huang employed Bodipy fluorescent dyes as acceptors and certain afterglow materials as donors to obtain a series of narrowband afterglow materials with a high color purity based on efficient Förster resonance energy transfer (FRET) [18]. Additionally, Zhang reported on a multi-resonance thermally activated delayed fluorescent (MRTADF) afterglow emitter, capable of achieving narrowband performance using TADF afterglow. Through a doping mechanism strategy, unprecedented long emission lifetimes exceeding 100 ms, an FWHM of less than 40 nm, and a CIEy deep-blue emission color of 0.048 were achieved [19]. However, the current narrowband FWHM does not reach the desired smallness, as it is consistently influenced by the structural relaxations of the excited states and vibration couplings between the ground and excited states. Furthermore, achieving the tunability of narrowband colors and a longer-wavelength emission in pure organic systems remains challenging.

Currently, the structural design and strategies of afterglow materials are continuously advancing. In recent years, the predominant molecular design strategies have revolved around heavy atom effects (HAEs) [20,21,22,23,24], aggregation state control [11,25,26,27,28], supramolecular assembly [7,29,30,31], and other approaches [32,33,34,35]. Additionally, high-performance materials have been successfully developed through two-component/multi-component technology. For instance, Kim’s group has achieved a high phosphorescence efficiency via cocrystal design [20]. Adachi and Hirata have reported long phosphorescence lifetimes of luminescent dopants in rigid steroid matrices [36]. Dong, Cai, and Lei have developed the fabrication of afterglow materials via the mediation of the matrix’s T_1_ states [37]. Meanwhile, Huang and coworkers have demonstrated a series of polymer-based narrowband afterglow materials utilizing the phosphorescence FRET mechanism [17]. Zhang’s group has revealed that the dipole–dipole interaction between the matrix and dopant’s S_1_ states can facilitate intersystem crossing (ISC) for constructing high-performance RTP and thermally activated delayed fluorescence (TADF) afterglow materials [38]. Moreover, there has been an extensive exploration of afterglow materials with intriguing functionalities, such as the sonication-responsive organic afterglow in an aqueous medium [39], TADF-type afterglow material as a time-gated chemical dosimeter [40], and others [41,42,43,44].

The simple and highly adaptable preparation of a two-component/multi-component strategy allows for the creation of new performance materials through the synergistic effect between the components in multi-component materials [45,46]. Here, an innovative approach based on a multi-component strategy on the integration of inorganic narrowband materials with organic long afterglow materials is reported to produce a series of color-tunable hybrid narrowband afterglow materials that can be excited by visible light.

In 2023, the Nobel Prize in Chemistry was awarded to Moungi G. Bawendi, Louis E. Brus, and Alexei I. Ekimov for their groundbreaking discovery and synthesis of QDs. QDs exhibit a high photoluminescence quantum yield (PLQY), narrow FWHM (<30 nm), and adjustable emission wavelength. However, their lifetime typically falls within the nanosecond range and lacks afterglow characteristics. For instance, CdSe QDs are well-suited for biological imaging applications [15,47,48,49,50,51]. To enhance their PLQY, the inorganic CdSe QDs are encapsulated by a layer of ZnS to prevent nonradiative decay. In the present study, several CdSe/ZnS QDs with different emission wavelengths (λ_QDs_) were selected as acceptors, combined with a two-component long organic afterglow material composed of Cor and 4-methoxybenzene (MeOBP) as donors. Through the hybridization of organic and inorganic methods, a three-component narrowband organic afterglow material with an adjustable color has been developed, showing great promise for various applications.

## 2. Results and Discussion

### 2.1. Cor-MeOBP Material Fabrication and Photophysical Measurements

**Figure 1:**
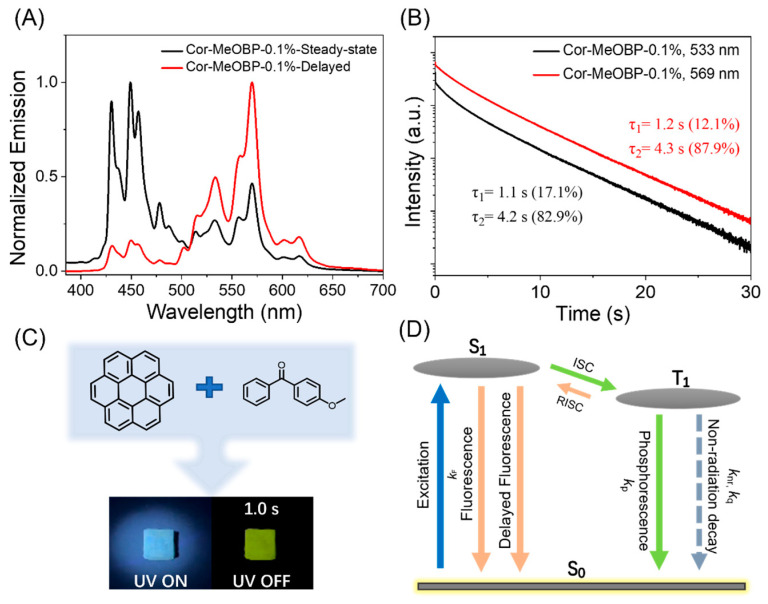
(**A**) Room-temperature steady-state and delayed emission (1 ms delay) spectra of Cor-MeOBP-0.1% sample. (**B**) Room-temperature emission decay of Cor-MeOBP-0.1% sample monitored at 533 nm and 569 nm, respectively. (**C**) Chemical structures of Cor and organic matrices in the present system and photographs of Cor-MeOBP-0.1% melt-cast samples under 365 nm UV lamp and upon ceasing UV lamp. (**D**) The proposed mechanism of organic afterglow in the Cor-matrix system at room temperature.

Cor molecules in the solution state and solid state do not show a room-temperature afterglow, possibly due to aggregation-caused quenching (ACQ). When Cor molecules are dispersed into either a phenyl benzoate (PhB) or MeOBP matrix, the resultant Cor-PhB and Cor-MeOBP samples exhibit an afterglow under ambient conditions (Figure S1 and Figure 1C). In the control experiment, neither the PhB matrix nor MeOBP matrix shows a room-temperature afterglow. Here, the matrix can disperse Cor molecules to avoid ACQ and protect Cor’s triplet excited states by a rigid crystalline environment, leading to a room-temperature afterglow. It is found that MeOBP is an exceptional organic matrix for producing highly luminescent Cor organic afterglow materials in ambient conditions [52,53,54,55] (here, the Cor-MeOBP sample exhibits a brighter afterglow than Cor-PhB), so we use MeOBP as the matrix in the present study for material fabrication. To prepare Cor-MeOBP-0.1%, 100 μL Cor in dichloromethane (1 mg/mL) and 100 mg MeOBP were added into an agate mortar (diameter = 5 cm) for grinding. Dichloromethane was added to assist the mixing of Cor and MeOBP. After grinding, followed by solvent evaporation and melt casting, Cor-MeOBP-0.1% samples at room temperature that show the afterglow property were obtained. It has been found that two-component Cor-MeOBP samples at different doping concentrations (0.01 wt%, 0.1 wt%, 1 wt%, 3 wt%, and 5 wt%) also exhibited a bright green organic afterglow under ambient conditions (Figures S2–S5). The steady-state emission spectra of Cor-MeOBP-0.1% materials show fluorescence plus phosphorescence dual emission with the fluorescence band in the range of 420 nm to 480 nm and the phosphorescence band in the range of 500 nm to 640 nm (Figure 1A). The existence of the phosphorescence band in the steady-state emission spectra suggests a high tendency of intersystem crossing in the system; the symmetry-forbidden property of S_1_-to-S_0_ emission in the Cor system reduces the fluorescence decay rate and, consequently, increases the intersystem crossing yield [54,55]. The delayed emission spectra (1 ms delay) exhibit a phosphorescence band ranging from 500 nm to 640 nm with a long phosphorescence lifetime of up to 4.3 s monitored at 569 nm (Figure 1B). The long phosphorescence lifetime indicates the small phosphorescence decay rate (*k*_P_) of Cor and the excellent protection of organic triplets in the MeOBP matrix. It is interesting to note that there is a minor delayed fluorescence band in the 420 nm to 480 nm region of the delayed emission spectra (Figure 1A). Variable delayed emission measurements show that the delayed fluorescence band increases with temperature (Figure S6). Given that the triplet–triplet annihilation at a low doping concentration such as 0.1% would be insignificant, the delayed fluorescence can be assigned to the thermally activated delayed fluorescence (TADF). Although the Cor system has a relatively large singlet–triplet splitting energy and, thus, has a relatively small rate constant of reverse intersystem crossing (RISC), the small *k*_RISC_ is still enough to open the TADF pathway in the Cor-MeOBP system featuring a small *k*_P_ and very small *k*_nr_ and *k*_q_ (rate constants of nonradiative decay and oxygen quenching). The reported studies of Cor systems [54,55], as well as our recent studies on TADF-type afterglow materials [38], support these assignments. As illustrated in Figure 1D, Cor molecules dispersed in the MeOBP matrix can be excited by UV or visible light to form singlet excited states, undergo intersystem crossing to reach triplet excited states, and, subsequently, emit ultralong RTP due to the small *k*_P_ of Cor and very small *k*_nr_ + *k*_q_ resulting from the rigid environment provided by the MeOBP matrix. Moreover, Cor’s triplet excited states can also undergo RISC to form singlet excited states, and then emit TADF under ambient conditions (Figure 1D).

### 2.2. Cor-MeOBP-QDs Material Fabrication

**Figure 2:**
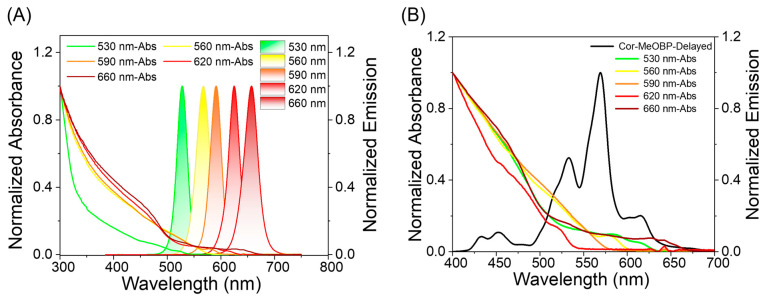
(**A**) UV–Vis absorption and steady-state emission spectra of five different wavelength QDs dispersions in n-hexane and absorption spectra of the QDs. (**B**) Delayed emission (1 ms delay) spectrum of Cor-MeOBP-0.1% sample (black line) and absorption spectra of the QDs.

In view of its long and bright afterglow and broad phosphorescence band (Figure 1A), the two-component Cor-MeOBP system may be an ideal donor for energy transfer. In order to prepare the narrowband afterglow materials, the innovative combination of inorganic CdSe/ZnS QDs with organic RTP materials is proposed. Figure 2A shows the narrowband emission and absorption spectra of a series of QDs. The absorption of the QDs and the delayed emission spectra of the Cor-MeOBP afterglow materials show a nice match (Figure 2B). By doping QDs of different concentrations or emission wavelengths into the Cor-MeOBP system, three-component narrowband long-lived organic afterglow materials are obtained.

### 2.3. Photophysical Property of Cor-MeOBP-QDs Materials

**Figure 3:**
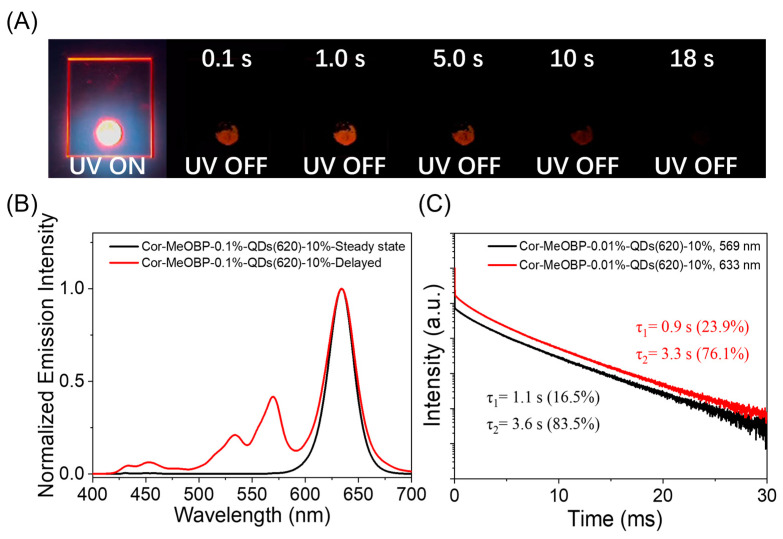
(**A**) Photographs of Cor-MeOBP-0.1%-QDs(620) three-component afterglow material under a UV lamp and upon ceasing UV excitation. (**B**) Room-temperature steady-state and delayed emission (1 ms delay) spectra and (**C**) room-temperature emission decay of Cor-MeOBP-0.1%-QDs(620) three-component system monitored at 569 nm and 633 nm, respectively.

For the preparation of three-component afterglow materials, firstly, we tested the stability of QDs during grinding. The emission spectra of the QD solution show an insignificant change before and after grinding (Figure S7). Then, we doped red QDs with an emission wavelength of 620 nm into the Cor-MeOBP-0.1% system. The resultant Cor-MeOBP-0.1%-QDs(620) materials exhibited a visible afterglow of up to 18 s (Figure 3A) and a delayed emission band at 633 nm with an FWHM of 36 nm at room temperature (Figure 3B). It is known that the entrance of scattered fluorescence light into the spectrograph slit during the illuminating phase of the phosphoroscope rotation may have some contribution to the delayed emission spectra. In the control experiment, the MeOBP-QDs(620) sample gives a very weak signal at 633 nm in the delayed emission spectra (Figure S8), so the scattered fluorescence light cannot explain the significant delayed emission band at 633 nm (Figure 3B). Moreover, the phosphorescence signals of Cor at 633 nm should be relatively weak, so it cannot explain the presence of the significant delayed emission band at 633 nm. In the case of Cor-MeOBP-QDs(660), we can find the significant delayed emission band at 675 nm with long emission lifetimes (Figure S9); the phosphorescence signals of Cor are negligibly small at 675 nm. Moreover, the emission decay profiles (Figure 3C) of Cor-MeOBP-0.1%-QDs(620) show that the emission intensity at 633 nm is stronger than that at 569 nm at any specific time points in the region from several milliseconds to several tens of seconds. These experiments and analysis confirm that the significant narrowband delayed emission can be exclusively assigned as the afterglow of QDs from Cor’s energy transfer. The emission lifetime of the Cor-MeOBP-0.1%-QDs(620) monitored at 633 nm can be fitted into the double-exponential decay with τ_1_ = 0.9 s (23.9%) and τ_2_ = 3.3 s (76.1%) (Figure 3C). Similarly, other three-component organic afterglow materials of the Cor-MeOBP system with different doping concentrations of QDs(620) also show a narrowband emission (Figure S10). Furthermore, three-component narrowband afterglow materials can also be prepared by using QDs of different wavelengths (Table 1). For example, by doping orange QDs with a wavelength of 590 nm into the Cor-MeOBP system, the obtained Cor-MeOBP-0.01%-QDs(590) and Cor-MeOBP-3%-QDs(590) exhibited delayed emission bands at 606 nm and 605 nm with an FWHM of 37 nm and 34 nm at room temperature, respectively (Figure S11). When the QDs wavelength of 660 nm was used, the Cor-MeOBP-0.01%-QDs(660) and Cor-MeOBP-3%-QDs(660) at room temperature show an FWHM of 48 nm and 44 nm, respectively (Figure S9). Interestingly, it is found that Cor-MeOBP-QDs materials can be excited by visible light such as 405 nm purple light, and 420 nm and 430 nm blue light, exhibiting a narrowband organic afterglow under environmental conditions (Figure S12). 

### 2.4. Photophysical Mechanism in Cor-MeOBP-QDs Narrowband Afterglow System

**Figure 4:**
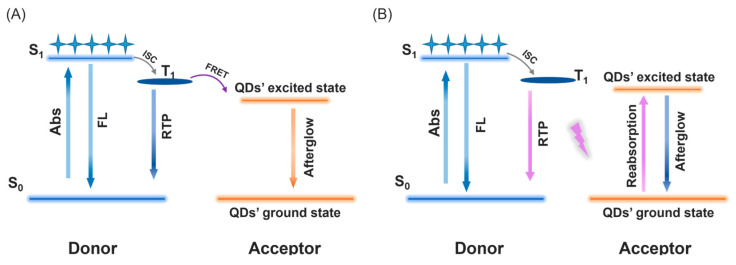
(**A**) Schematic illustration of excited state energy transfer from donor’s T_1_ to acceptor’s S_1_. (**B**) Reabsorption mechanism where the fluorescence acceptor absorbs donor’s afterglow lights.

In the reported organic afterglow systems, there are mainly two types of energy transfer mechanisms [56,57,58,59,60,61]: (1) the excited state energy transfer from the afterglow donor to the fluorescence acceptor, and (2) the reabsorption mechanism; that is, the fluorescence acceptor can absorb the donor’s afterglow light, be excited, and then show a fluorescence afterglow (Figure 4). To investigate the energy transfer mechanism, we first perform the measurement of the fluorescence decay of the Cor-MeOBP and Cor-MeOBP-QDs samples. Figure S13 shows that Cor’s fluorescence lifetime undergoes a drastic decrease in the Cor-MeOBP-QDs sample, while the QDs fluorescence lifetime shows an increase in the Cor-MeOBP-QDs sample. These observations suggest the fluorescence energy transfer from Cor to QDs, demonstrating the communication between Cor and QDs in the three-component system. Then, the afterglow decay profiles at 569 nm (Cor’s phosphorescence maximum) have also been monitored. The afterglow lifetimes of the Cor-MeOBP and Cor-MeOBP-QDs samples have been summarized in Table 2. It has been found that the afterglow lifetime of the Cor-MeOBP-QDs sample at 569 nm is shorter than that of the corresponding Cor-MeOBP sample. With reference to the reported studies [56,57,58,59,60,61], such a lifetime decrease suggests the excited state energy transfer from Cor’s T_1_ states to QDs. The afterglow energy transfer efficiency (Φ_ET_, Table 2) can be estimated by the change in Cor’s afterglow lifetimes based on the reported equation [54,58,61]. When compared to the fluorescence energy transfer in the Cor-MeOBP-QDs system, the change in the afterglow lifetime is less drastic than that in fluorescence lifetime (Figure S13). This can be explained by the observation that the spectral overlap between Cor’s fluorescence and QDs’ absorption is better than that between Cor’s phosphorescence and QDs’ absorption, as well as the spin-forbidden and spin-allowance nature in the system. Currently, a moderate efficiency of the afterglow energy transfer can be achieved. The further enhancement of the afterglow efficiency is still in progress. It is known that QDs themselves do not have an afterglow property. In the case of MeOBP-QDs (Figure S8), the sample also does not show an afterglow property. In the Cor-MeOBP-QDs systems, upon the afterglow energy transfer, the afterglow lifetime of QDs has been found to be similar to Cor’s afterglow lifetime (Table 2). With reference to the reported afterglow systems [54,59], it is common that, after the energy transfer, the fluorescence acceptor has a similar afterglow lifetime to the afterglow donor. Here, the observation (Table 2) agrees well with the reported studies. Therefore, the excited energy transfer from the Cor’s T_1_ states to QDs should be responsible for the emergence of the narrowband afterglow in the present study (Figure 4A). For the reabsorption mechanism (Figure 4B), due to the relatively low absorbance of QDs in Cor’s phosphorescence region, the efficiency would be low. For the illustration of the excited state energy transfer, the Jablonski diagram is preferred in the reported studies, so we illustrate the proposed mechanism in Figure 4B. Moreover, we also present the HOMO and LUMO distribution of the Cor and the electron-hole iso-surface maps of Cor’s singlet excited states and triplet excited states were calculated by the TD-DFT method (Figures S14 and S15). The size-dependent valence band and conduction band of QDs can be found in the reported study [62].

### 2.5. TBA-DA-QDs Material Fabrication and Photophysical Measurements

**Figure 5:**
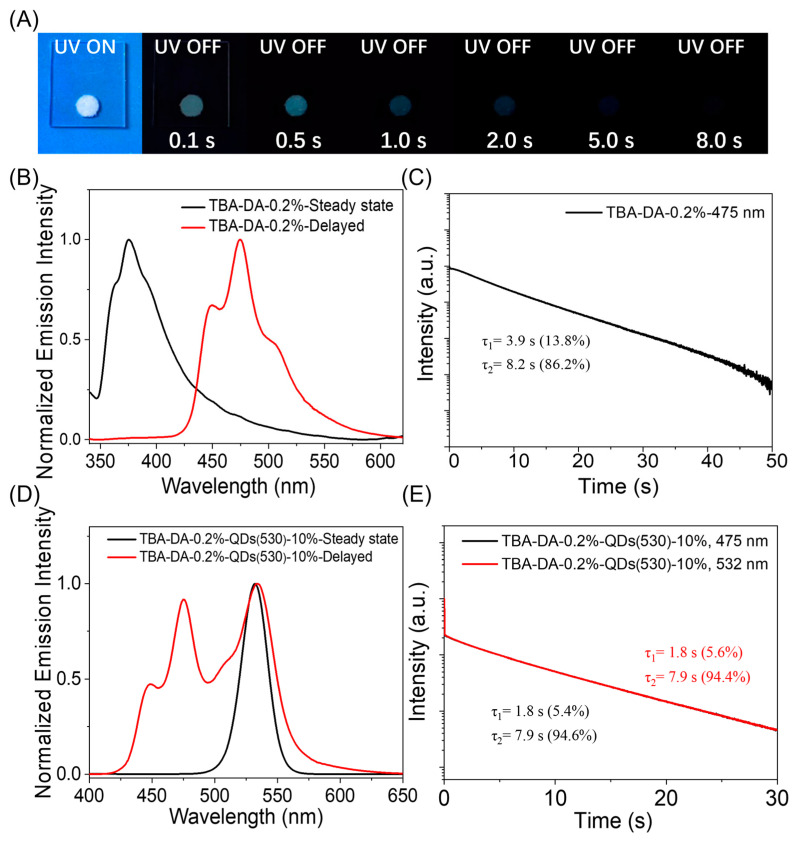
(**A**) Photographs of TBA-DA-0.2% two-component afterglow material under a UV lamp and upon ceasing UV excitation. (**B**) Room-temperature steady-state and delayed emission (1 ms delay) spectrum and (**C**) emission decay monitored at 475 nm of TBA-DA-0.2%-QDs(530). (**D**) Room-temperature steady-state and delayed emission (1 ms delay) spectrum and (**E**) emission decay monitored at 475 and 532 nm of TBA-DA-QDs(530) three-component system.

Similarly, we also used the polyaromatic compound triphenylboronic acid (TBA) doped into a sebacic acid (DA) matrix, and, by doping QDs, a three-component narrowband long afterglow material can also be prepared. Firstly, the two-component materials of TBA-DA-0.2% exhibited a fluorescence band in the range of 350 nm to 420 nm in their steady-state emission spectra and a phosphorescence band ranging from 450 to 520 nm in the delayed emission spectrum (1 ms delay) and a long phosphorescence lifetime of up to 8.2 s (Figure 5A,B). The absorption measurements of the QDs and the delayed emission spectra of the TBA-DA afterglow materials also showed a good match (Figure S16). Doping QDs with wavelengths of 530 nm, 560 nm, and 620 nm into TBA-DA systems can also prepare TBA-DA-QDs three-component organic narrowband long afterglow materials (Figure S17). TBA-DA-QDs(530) exhibited delayed emission bands of 532 nm at room temperature (Figure 5C,D) and the phosphorescence lifetime is 7.9 s. TBA can also serve as a donor for a long afterglow, but, due to the low overlap between its emission spectrum and the absorption of QDs, the energy transfer efficiency is not better than that of Cor-MeOBP-QDs. Therefore, it further confirms the mechanism of three-component narrowband organic afterglow generation.

### 2.6. Material Functions

**Figure 6:**
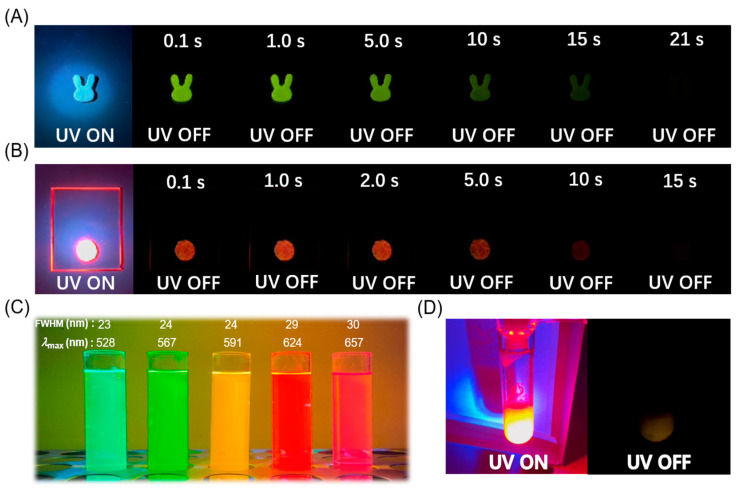
(**A**) Rabbit-shaped afterglow pattern of Cor-MeOBP-0.1% obtained by UV excitation. (**B**) Photographs of circular Cor-MeOBP-QDs(620) three-component afterglow material under an UV lamp and upon ceasing UV excitation. (**C**) Photographs demonstrating the size-tunable fluorescence properties of the five QDs dispersions in n-hexane. (**D**) Photographs of Cor-PMMA-QDs(620) emulsion under 365 nm UV and after ceasing the UV lamp at room temperature.

Red organic afterglow materials with a bright and long-life emission have high value in enriching afterglow colors and biological imaging applications. In view of their long afterglow lifetimes and the excellent processability, the Cor-MeOBP-QDs materials are selected for the demonstration of the functions of afterglow materials. The Cor-MeOBP materials can be readily processed into rabbit-shaped objects by melt casting with the aid of silicone molds (Figure 6A). The pattern of the circular afterglow is obtained by Cor-MeOBP-QDs in a quartz mold (Figure 6B). QDs at different wavelengths display different colors under UV irradiation (Figure 6C), which can be used to prepare three-component wavelength-adjustable organic afterglow materials. The excellent processablity into desired shapes of Cor-MeOBP-QDs systems would endow them with the application potential in high-quality anti-counterfeiting. However, the Cor-MeOBP-QDs systems lack aqueous dispersity. To further explore the function of the narrowband organic afterglow system, here, we try the preparation of aqueous afterglow materials. It is known that, most existing methods for producing aqueous afterglow materials rely on the mechanical processing of solid-state afterglow materials, which can disrupt the protective environment for organic triplets and lead to a significant loss of afterglow performance. In this study, we incorporate Cor and QDs with a wavelength of 620 nm into an emulsion polymerization system to create an afterglow dispersion (Figure 6D). The resulting Cor-PMMA-QDs emulsion exhibits a delayed emission band at 624 nm with an afterglow lifetime of 2.5 s (Figure S18). The afterglow lifetime at Cor’s phosphorescence maximum is 3.4 s, while the phosphorescence lifetime of the Cor-PMMA afterglow emulsion (Figure S19) at 567 nm is 4.5 s. The Φ_ET_ in the Cor-PMMA-QDs emulsion system can be estimated to be 24.4%. The Cor-PMMA-QDs emulsion exhibits an excellent aqueous dispersity, which holds promise for biological imaging applications in aqueous media. 

## 3. Materials and Methods

### 3.1. Materials 

The following materials were used: Coronene (Cor) (98%, Zhengzhou Alpha Chemical Co., Zhengzhou, China), 4-methoxybenzophenone (MeOBP) (99%, Adamas, Shanghai, China), phenyl benzoate (PhB) (99%, Energy Chemical, Shanghai, China), quantum dot CdSe/ZnS (QDs) (Xiamen BOHR Technology Co., Xiamen, China), triphenylen-2-ylboronic acid (TBA) (98%, Shanghai D&B Biological Science and Technology Co., Ltd., Shanghai, China), 1,10-decanedioic acid (DA) (95%, Bide Pharmatech Ltd., Shanghai, China), methyl methacrylate (MMA) (99%, Adamas), potassium persulfate (KPS) (98%, Innochem, Beijing, China), and Pluronic F127 (poly(ethylene oxide)-*b*-poly(propylene oxide)-*b*-poly(ethylene oxide), PEO_100_-*b*-PPO_68_-*b*-PEO_100_; the subscript represents the degree of polymerization of each block, Sigma-Aldrich, St. Louis, MO, USA. The photophysical properties of the five different types of CdSe/ZnS QDs provided by the company are consistent with our measurement (Table S1). 

### 3.2. Physical Measurements and Instrumentation

UV–Vis absorption spectra were recorded on a Shimadzu UVmini-1285 UV–Vis spectrophotometer (Kyoto, Japan). The steady-state and delayed emission spectra were collected by Hitachi FL-4700 fluorescence spectrometer (Tokyo, Japan) equipped with chopping systems; the delayed emission spectra were obtained with a delay time of approximately 1 ms. The excited state decay profiles in millisecond to second region were collected by Hitachi FL-4700 fluorescence spectrometer equipped with chopping systems. The fluorescence decay profiles in nanosecond region were recorded by using time-correlated single photon counting (TCSPC) technique on an Edinburgh FLS1000 fluorescence spectrometer (Edinburgh Instruments Ltd., Livingston, UK) equipped with a picosecond pulsed diode laser. Photographs and videos were captured by iPhone 13 camera (Apple Campus, Cupertino, CA, USA). Before the capture, samples were irradiated by a 365 nm UV lamp (5 W) for approximately 5 s at approximately 15 cm. 

### 3.3. Preparation of the Two-Component Afterglow System

To prepare Cor-MeOBP-0.1%, 100 μL Cor in dichloromethane (1 mg/mL) and 100 mg MeOBP were added into an agate mortar (diameter = 5 cm) for grinding. Dichloromethane was added to assist the mixing of Cor and MeOBP. After grinding, followed by solvent evaporation and melt casting, the Cor-MeOBP-0.1% that shows afterglow property was obtained. Other afterglow materials with different doping concentrations in this study were prepared through similar processes.

To prepare TBA-DA-0.2%, 200 μL TBA in dichloromethane (1 mg/mL) and 100 mg DA were added into an agate mortar (diameter = 5 cm) for grinding. Dichloromethane and a little ethanol were added to assist the mixing of TBA and DA. After grinding, followed by solvent evaporation, the TBA-DA-0.2% that shows afterglow property was obtained. Other afterglow materials with different doping concentrations in this study were prepared through similar processes.

### 3.4. Preparation of Three-Component Afterglow Materials by Doping QDs into Afterglow System

Cor-MeOBP-QDs three-component afterglow materials were prepared by doping Cor and QDs into MeOBP with different doping concentrations. For example, 20 μL Cor dichloromethane solution (1 mg/mL), 20 mg MeOBP, and 132 μL CdSe/ZnS in n-hexane solution (15 mg/mL) were added to an agate mortar (diameter = 5 cm) for grinding. The obtained powders were then processed by melt casting to form Cor-MeOBP-0.1%-QDs(10%) with a doping concentration of 0.1% Cor and 10% QDs of MeOBP. Other Cor-MeOBP-QDs three-component afterglow materials were prepared by the similar procedures.

TBA-DA-QDs three-component afterglow materials were prepared by doping TBA and QDs into DA with different doping concentrations. For example, 40 μL TBA dichloromethane solution (1 mg/mL), 20 mg DA, 132 μL CdSe/ZnS in n-hexane solution (15 mg/mL), and a little ethanol were added to an agate mortar (diameter = 5 cm) for grinding. The obtained powders after solvent evaporation were TBA-DA-0.2%-QDs(10%) with a doping concentration of 0.2% TBA and 10% QDs of DA. Other TBA-DA-QDs three-component afterglow materials were prepared by the similar procedures.

### 3.5. Preparation of Cor-PMMA-QDs Organic Afterglow Emulsions

The Cor was first dissolved in MMA at a concentration of 0.1 mg/mL. Then, 100 μL Cor/MMA solution, 120 μL MMA, 1 mL of Pluronic F127 surfactant in aqueous solution (25 mg/mL), 2 mL deionized water, and 200 μL CdSe/ZnS/n-hexane solution (1 mg/mL) were added into a 10 mL Schlenk tube. The liquid precursor was treated by 30 min ultrasonication for pre-emulsification, and then 0.5 mL of potassium persulfate (2 mg/mL) was added into the liquid precursor. After three cycles of freeze–pump–thaw–degassing procedures, the liquid precursor was treated by sonication, and then stirred at 80 °C for 1.5 h, leading to the formation of Cor-PMMA-QDs organic afterglow emulsions.

## 4. Conclusions

In conclusion, the present study demonstrates a new concept for the preparation of narrowband organic/inorganic hybrid afterglow materials through energy transfer technology. A three-component narrowband afterglow material was obtained by using inorganic QDs as an acceptor and organic afterglow materials with long phosphorescence characteristics and wide phosphorescence bands as donors for energy transfer. As a result of the small FWHM of QDs, the present narrowband afterglow system achieves an FWHM of 31 nm. The concept of organic/inorganic hybridization would have a significant impact in the field of organic afterglow materials. The materials in the present study would show potential applications in multi-level imaging and high-density information encryption.

## Figures and Tables

**Table 1:** Photophysical data of three-component narrowband afterglow materials.

QDs Wavelength/nm	Cor/Con-centration	QDs/Con-centration	λ/nm ^a^	FWHM/nm	τ/s ^b^
590	0.01%	10%	606	37	τ_1_ = 0.8 (11.7%) τ_2_ = 3.0 (89.3%)
	3%	15%	605	34	τ_1_ = 0.4 (4.6%) τ_2_ = 2.4 (95.4%)
620	0.01%	10%	636	33	τ_1_ = 0.6 (8.3%) τ_2_ = 2.6 (91.7%)
	0.1%	10%	633	36	τ_1_ = 0.9 (23.9%) τ_2_ = 3.3 (76.1%)
	1%	10%	636	31	τ_1_ = 1.1 (23.1%) τ_2_ = 3.4 (76.9%)
	3%	15%	638	37	τ_1_ = 0.8 (23.7%) τ_2_ = 2.7 (76.3%)
660	0.01%	10%	667	48	τ_1_ = 0.6 (15.3%) τ_2_ = 2.4 (84.7%)
	0.1%	10%	670	42	τ_1_ = 0.5 (5.1%) τ_2_ = 3.0 (94.9%)
	3%	15%	675	44	τ_1_ = 0.7 (20.4%) τ_2_ = 2.6 (79.6%)

^a^ emission maximum of QDs in the delayed spectra; ^b^ afterglow lifetime monitored at QDs’ emission maximum.

**Table 2:** Afterglow lifetimes of Cor-MeOBP and Cor-MeOBP-QDs materials and estimated energy transfer efficiency.

QDs Wavelength/nm	Cor/Concentration	Cor-MeOBP’s τ/s ^a^	QDs/Concentration	Cor-MeOBP-QDs’ τ/s ^a^		Φ_ET_
		at 569 nm		at 569 nm	at λ_QDs_	
590	0.01%	4.0	10%	3.0	2.8	25%
	3%	3.9	15%	2.6	2.3	33.3%
620	0.01%	4.0	10%	2.7	2.4	32.5%
	0.1%	3.9	10%	3.2	2.7	17.9%
	1%	4.1	10%	3.5	2.9	14.6%
	3%	3.9	15%	2.5	2.2	35.9%
660	0.01%	4.0	10%	2.3	2.1	42.5%
	0.1%	3.9	10%	2.9	2.7	25.6%
	3%	3.9	15%	2.2	2.5	43.6%

^a^ average afterglow emission lifetime.

## Data Availability

The data presented in this study are available upon request from the corresponding author.

## Supplementary Materials

The following supporting information can be downloaded at: https://www.mdpi.com/article/10.3390/molecules29102343/s1, Table S1: The photophysical properties of CdSe/ZnS QDs; Figures S1–S5: Photographs and spectra of two-component afterglow materials; Figure S6: Temperature dependent delay spectrum of Cor-MeOBP-0.1%; Figure S7: Spectra of CdSe/ZnS-620 in n-hexane solution; Figure S8: Photographs and spectra of QDs-MeOBP(620) two-component material; Figures S9–S13 and S20: Photographs and spectra of Cor-MeOBP-QDs three-component materials; Figure S14: The HOMO and LUMO of the Cor molecule; Figure S15: TD-DFT-calculated electron density difference of singlet and triplet excited states of Cor: Figures S16 and S17: Spectra of TBA-DA-QDs three-component materials; Figure S18: spectra of Cor-PMMA-QDs(620) emulsion; Figure S19: Photographs and spectra of Cor-PMMA emulsion.
