# Supplementary material for: Narrowband Organic/Inorganic Hybrid Afterglow Materials
Source: Molecules. 2024 May 16;29(10):2343. doi: 10.3390/molecules29102343 (PMC11123977; doi:10.3390/molecules29102343)
Supplement: Supplementary file 1 [file molecules-29-02343-s001.zip › molecules-2980241-supplementary.pdf]

Supporting Information

Narrowband Organic/Inorganic Hybrid Afterglow Materials

Wen Xia [1,2], Xun Li [2], Junbo Li [1,2], Qianqian Yan [2], Guangming Wang [2], Xixi Piao [2,*] and Kaka Zhang [1,2,*]

[1] College of Chemistry and Materials Science, Sichuan Normal University, Chengdu 610068, China; xiawen@sioc.ac.cn (W.X.); lijunbo@sioc.ac.cn (J.L.)

[2] State Key Laboratory of Organometallic Chemistry, Key Laboratory of Synthetic and Self-Assembly Chemistry for Organic Functional Molecules, Shanghai Institute of Organic Chemistry, University of Chinese Academy of Sciences, Chinese Academy of Sciences, 345 Lingling Road, Shanghai 200032, China; lixun@sioc.ac.cn (X.L.); yanqianqian@sioc.ac.cn (Q.Y.); gmw@sioc.ac.cn (G.W.)

* Correspondence: piaoxixi@sioc.ac.cn (X.P.); zhangkaka@sioc.ac.cn (K.Z.)

Table S1. The photophysical properties of CdSe/ZnS QDs.

Name	Emission Wavelength/ nm[a]	Emission Wavelength /nm[b]	FWHM/ nm[a]	FWHM /nm[b]	PLQY[a]	Solvent[a]
CdSe/ZnS-530	530±10 nm	528	≤26 nm	23	≥90%	hexane
CdSe/ZnS-560	560±10 nm	567	≤28 nm	29	≥80%	hexane
CdSe/ZnS-590	590±10 nm	591	≤28 nm	24	≥80%	hexane
CdSe/ZnS-620	620±10 nm	624	≤28 nm	24	≥90%	hexane
CdSe/ZnS-640	640±10 nm	642	≤28 nm	26	≥80%	hexane
CdSe/ZnS-660	660±10 nm	657	≤28 nm	30	≥80%	hexane

[a] from the Xiamen BOHR Technology Co; [b] measured in our lab.

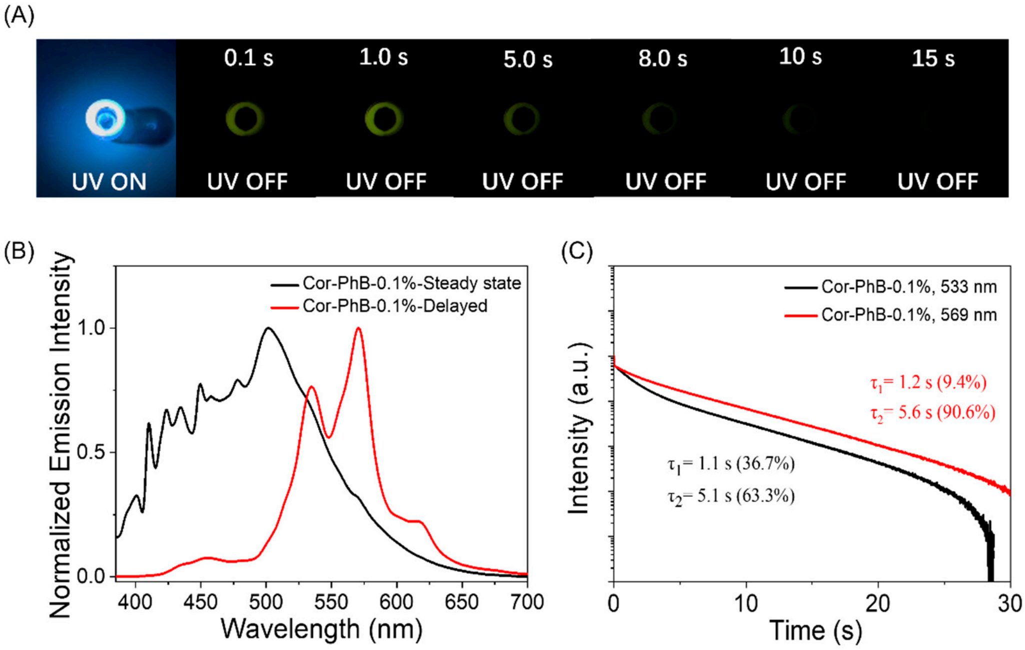

Figure S1. (A) Photographs of Cor-PhB-0.1% two-component afterglow material under an UV lamp and upon ceasing UV excitation. (B) Room-temperature steady-state (black line) and delayed emission (1 ms delay, red line) spectra and (C) emission decay profiles monitored at 533 and 569 nm of Cor-PhB-0.1% two-component system.

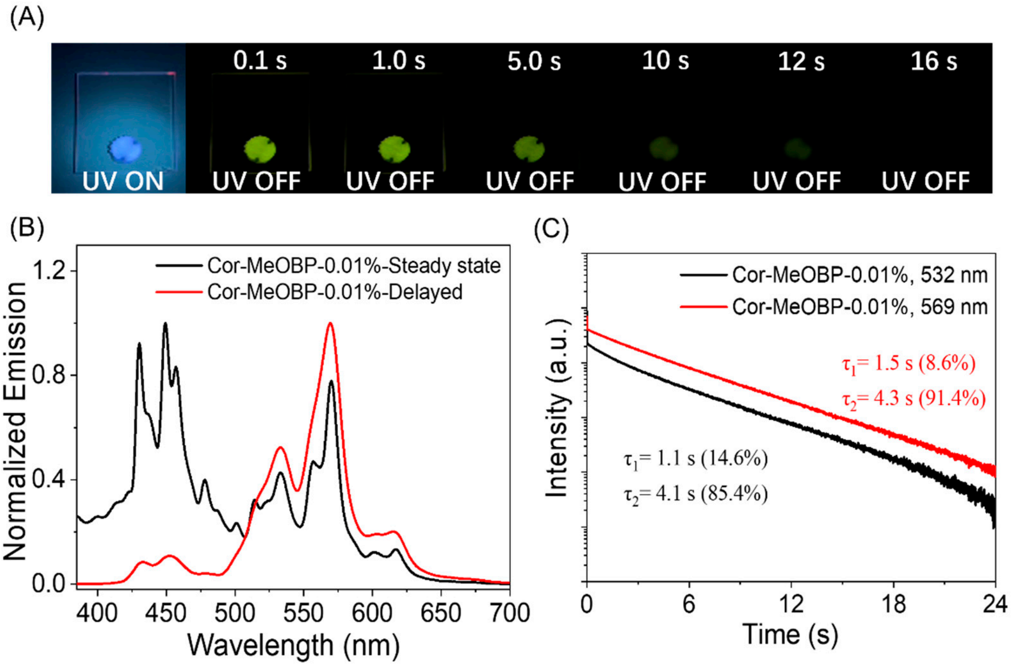

Figure S2. (A) Photographs of Cor-MeOBP-0.01% two-component afterglow material under an UV lamp and upon ceasing UV excitation. (B) Room-temperature steady-state (black line) and delayed emission (1 ms delay, red line) spectra and (C) emission decay profiles monitored at 532 and 569 nm of Cor-MeOBP-0.01% two-component system.

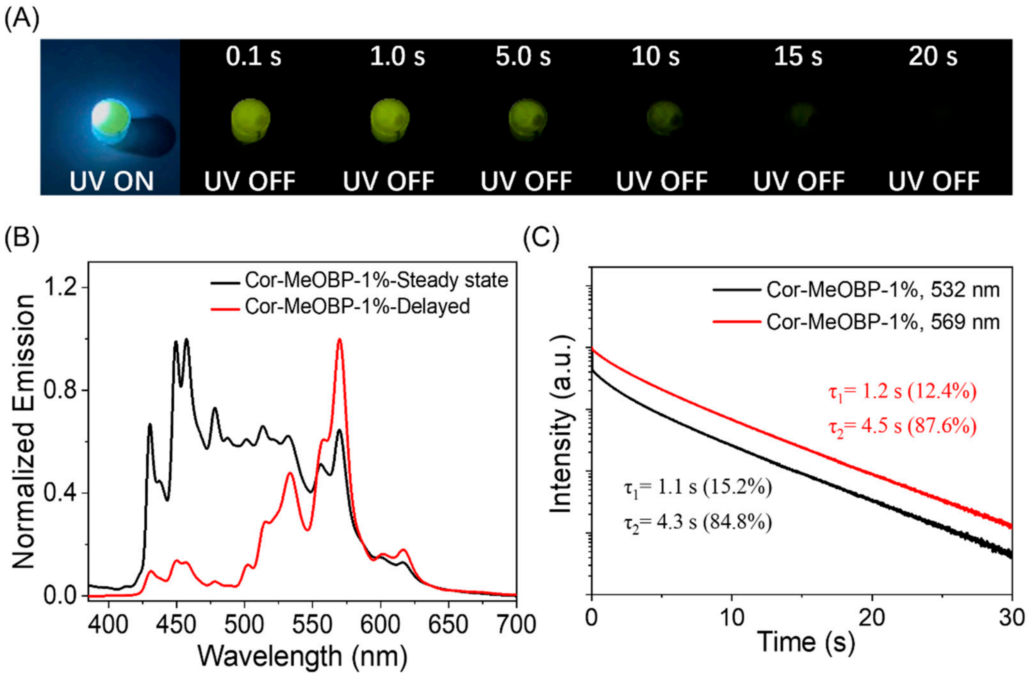

Figure S3. (A) Photographs of Cor-MeOBP-0.1% two-component afterglow material under an UV lamp and upon ceasing UV excitation. (B) Room-temperature steady-state (black line) and delayed emission (1 ms delay, red line) spectra and (C) emission decay profiles monitored at 532 and 569 nm of Cor-MeOBP-0.1% two-component system.

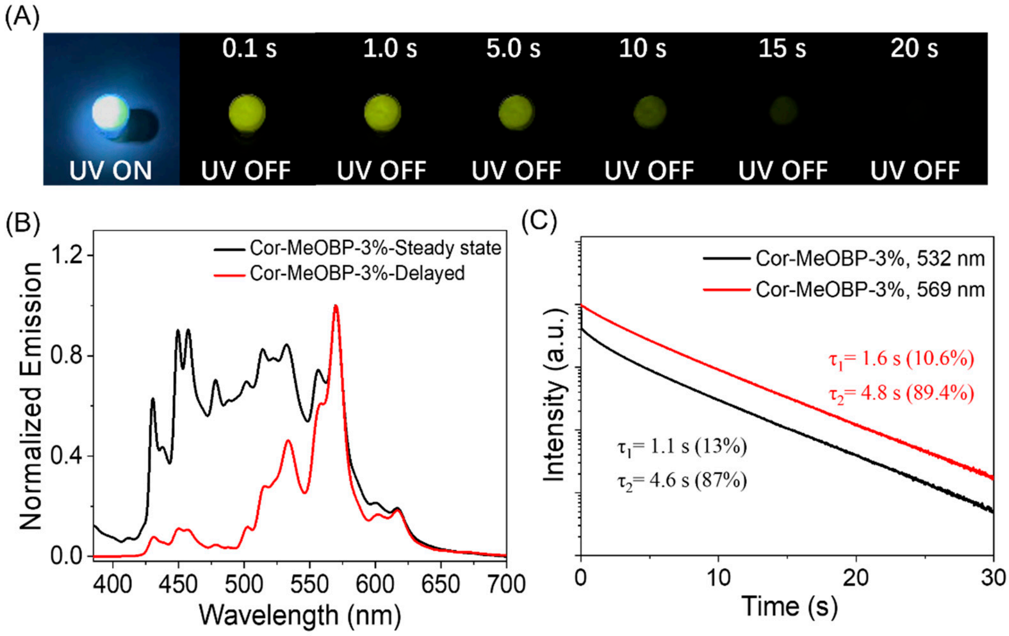

Figure S4. (A) Photographs of Cor-MeOBP-3% two-component afterglow material under an UV lamp and upon ceasing UV excitation. (B) Room-temperature steady-state (black line) and delayed emission (1 ms delay, red line) spectra and (C) emission decay profiles monitored at 532 and 569 nm of Cor-MeOBP-3% two-component system.

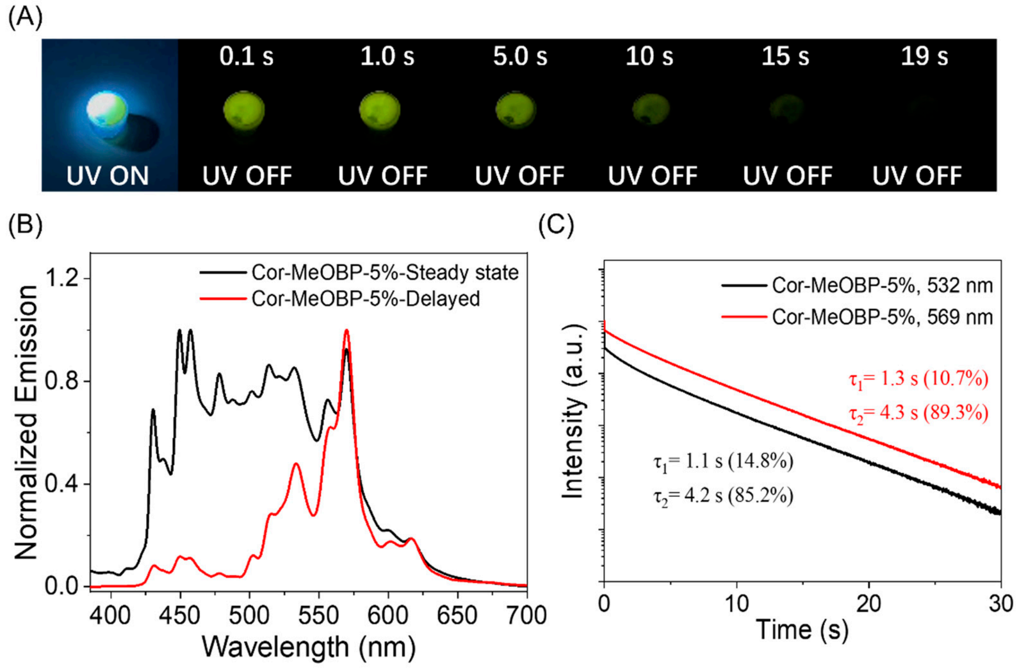

Figure S5. (A) Photographs of Cor-MeOBP-5% two-component afterglow material under an UV lamp and upon ceasing UV excitation. (B) Room-temperature steady-state (black line) and delayed emission (1 ms delay, red line) spectra and (C) emission decay profiles monitored at 532 and 569 nm of Cor-MeOBP-5% two-component system.

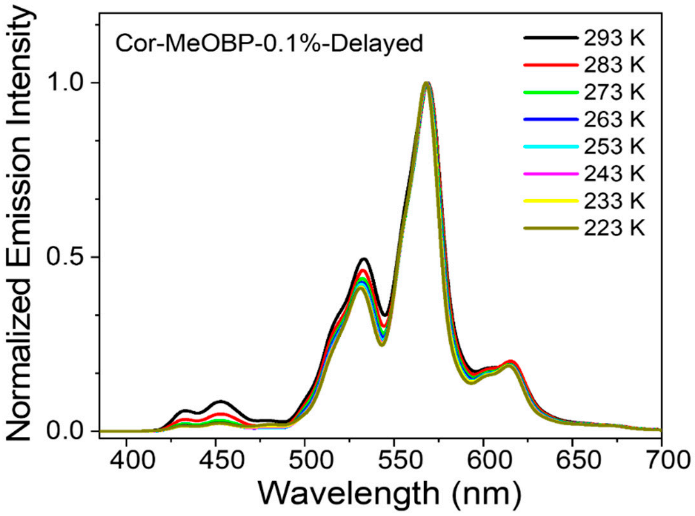

Figure S6. Variable temperature delayed emission (1 ms delay) spectra of Cor-MeOBP-0.1% material.

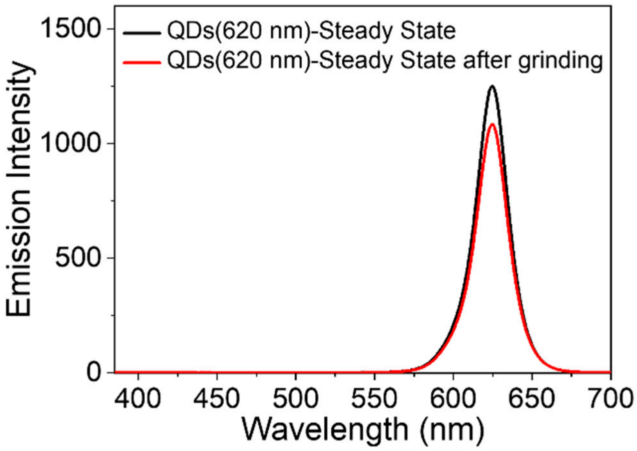

Figure S7. Room-temperature steady-state spectra before (black line) and after (red line) grinding of CdSe/ZnS-620 in n-hexane solution.

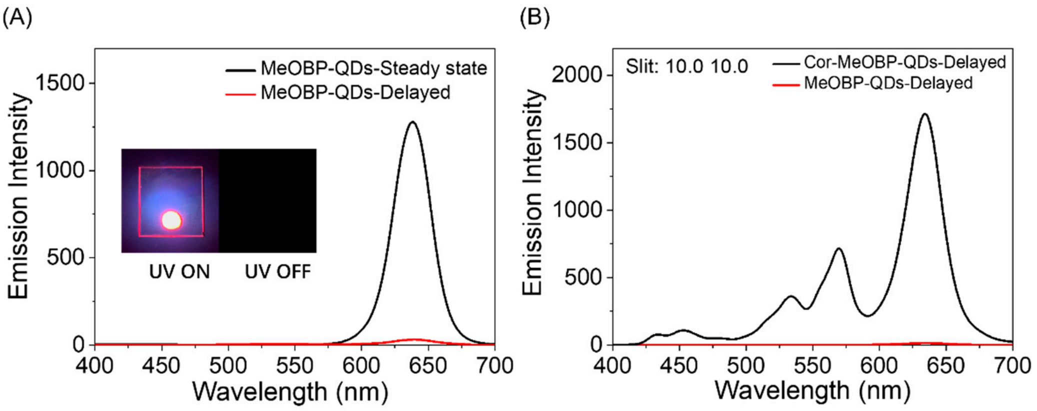

Figure S8. (A) Photographs of QDs-MeOBP(620) two-component material under an UV lamp (365 nm) and upon ceasing UV excitation and room-temperature steady-state emission spectra (black line) and delayed emission spectra (1 ms delay, red line) of MeOBP-QDs(620). (B) Room-temperature delayed emission spectra of Cor-MeOBP-0.1%-QDs(620)-10% (black line) and MeOBP-QDs(620) (red line).

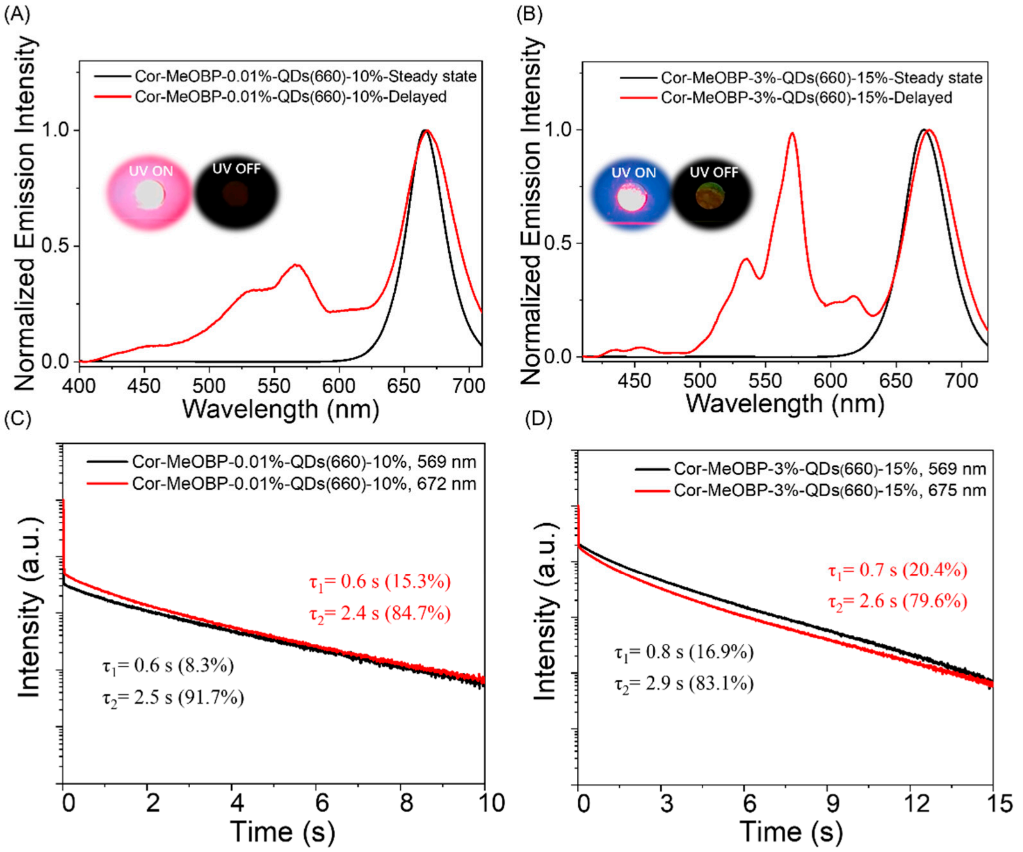

Figure S9. (A) Room-temperature steady-state (black line) and delayed emission (1 ms delay, red line) spectra and (C) emission decay monitored at 570 and 672 nm of Cor-MeOBP-0.01%-QDs(660) three-component afterglow material. (B) Room-temperature steady-state (black line) and delayed emission (1 ms delay, red line) spectra and (D) emission decay monitored at 569 and 675 nm of Cor-MeOBP-3%-QDs(660) three-component afterglow material.

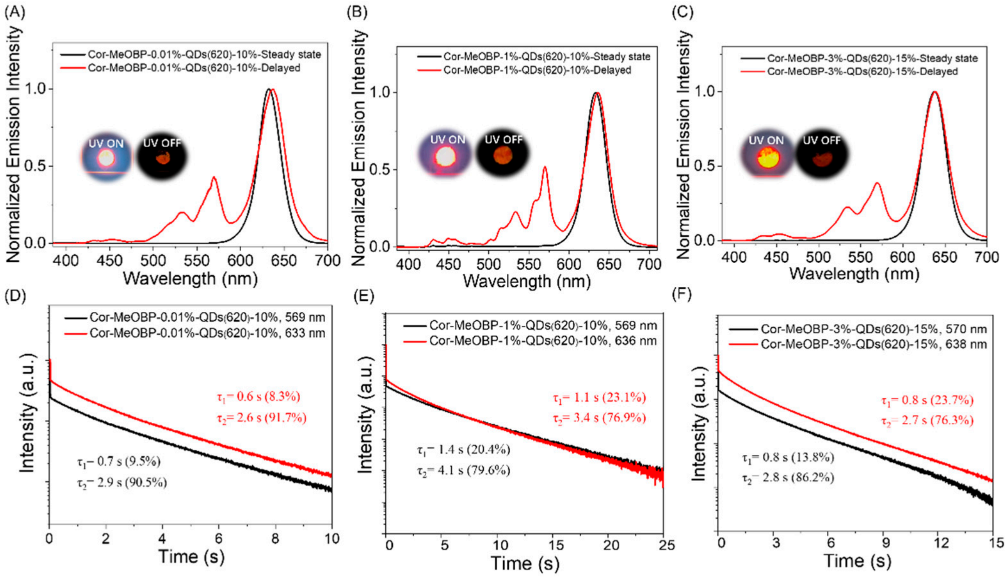

Figure S10. (A) Room-temperature steady-state (black line) and delayed emission (1 ms delay, red line) spectra and (D) emission decay monitored at 569 and 633 nm of Cor-MeOBP-0.01%-QDs(620) three-component afterglow material. (B) Room-temperature steady-state (black line) and delayed emission (1 ms delay, red line) spectra and (E) emission decay monitored at 569 and 636 nm of Cor-MeOBP-1%-QDs(620) three-component afterglow material. (C) Room-temperature steady-state (black line) and delayed emission (1 ms delay, red line) spectra and (F) emission decay monitored at 569 and 638 nm of Cor-MeOBP-3%-QDs(620) three-component afterglow material.

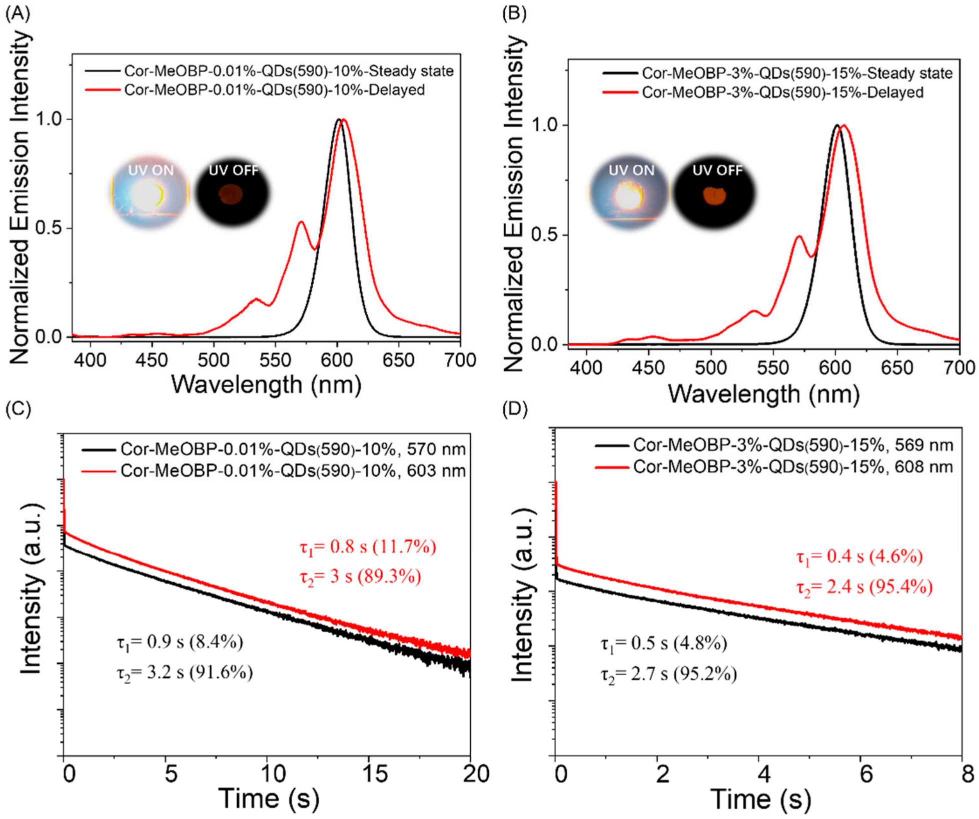

Figure S11. (A) Room-temperature steady-state (black line) and delayed emission (1 ms delay, red line) spectra and (C) emission decay monitored at 570 and 603 nm of Cor-MeOBP-0.01%-QDs(590) three-component afterglow material. (B) Room-temperature steady-state (black line) and delayed emission (1 ms delay, red line) spectra and (D) emission decay monitored at 569 and 608 nm of Cor-MeOBP-3%-QDs(590) three-component afterglow material.

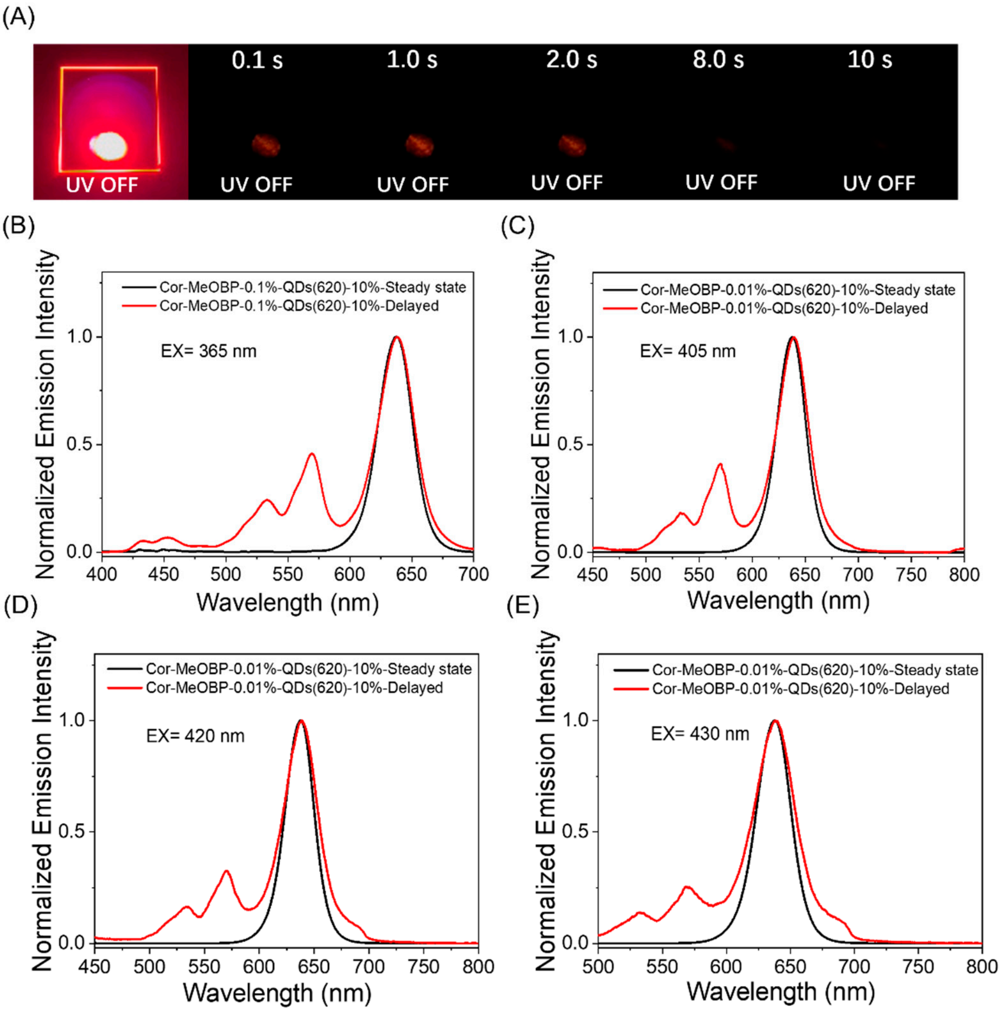

Figure S12. (A) Photographs of Cor-MeOBP-0.1%-QDs(620)-10% three-component afterglow material under an UV lamp (405 nm) and upon ceasing UV excitation. (B-E) room-temperature steady-state emission spectra (black line) and delayed emission spectra (1 ms delay, red line) of Cor-MeOBP-QDs(620) three-component materials excited at (B) 365 nm, (C) 405 nm, (D) 420 nm and (E) 460 nm, respectively.

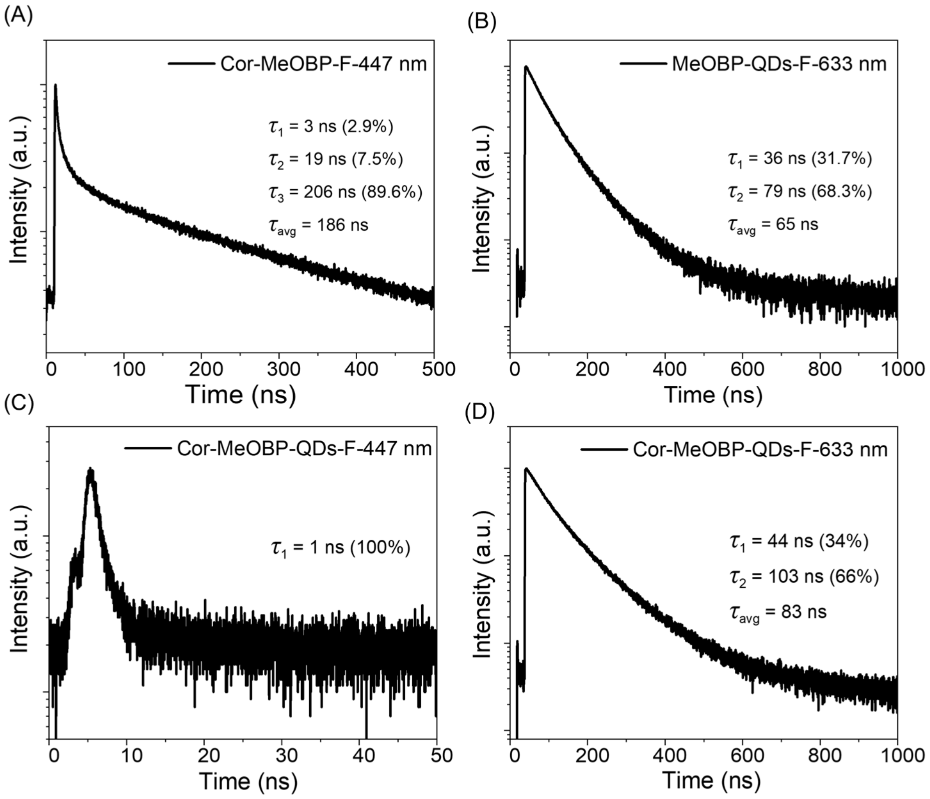

Figure S13. Fluorescence decay profiles of (A) Cor-MeOBP-0.1% at 447 nm, (B) MeOBP-QDs-10% at 633 nm, and Cor-MeOBP-0.1%-QDs(620)-10% at 447 nm (C) and 633 nm (D) respectively.

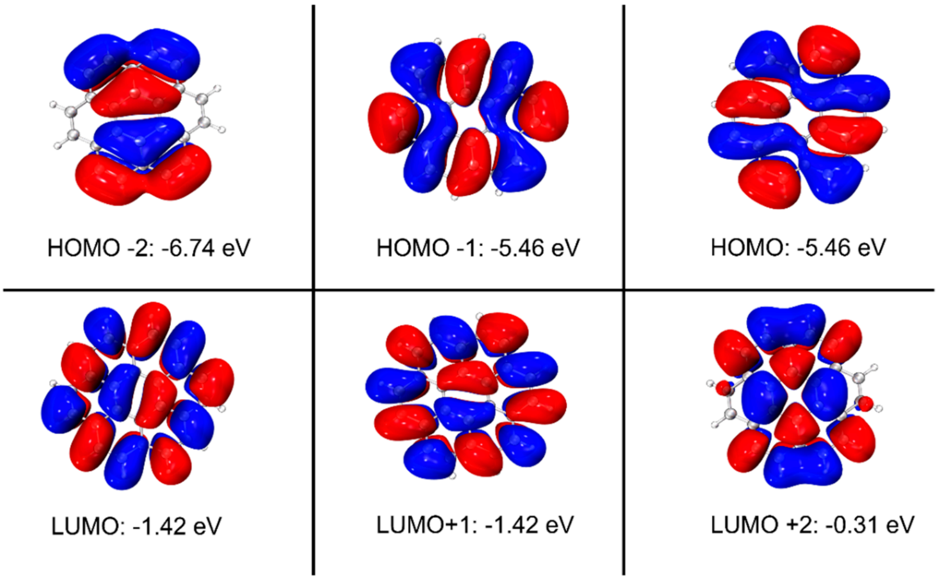

Figure S14. The HOMO and LUMO of the Cor molecule.

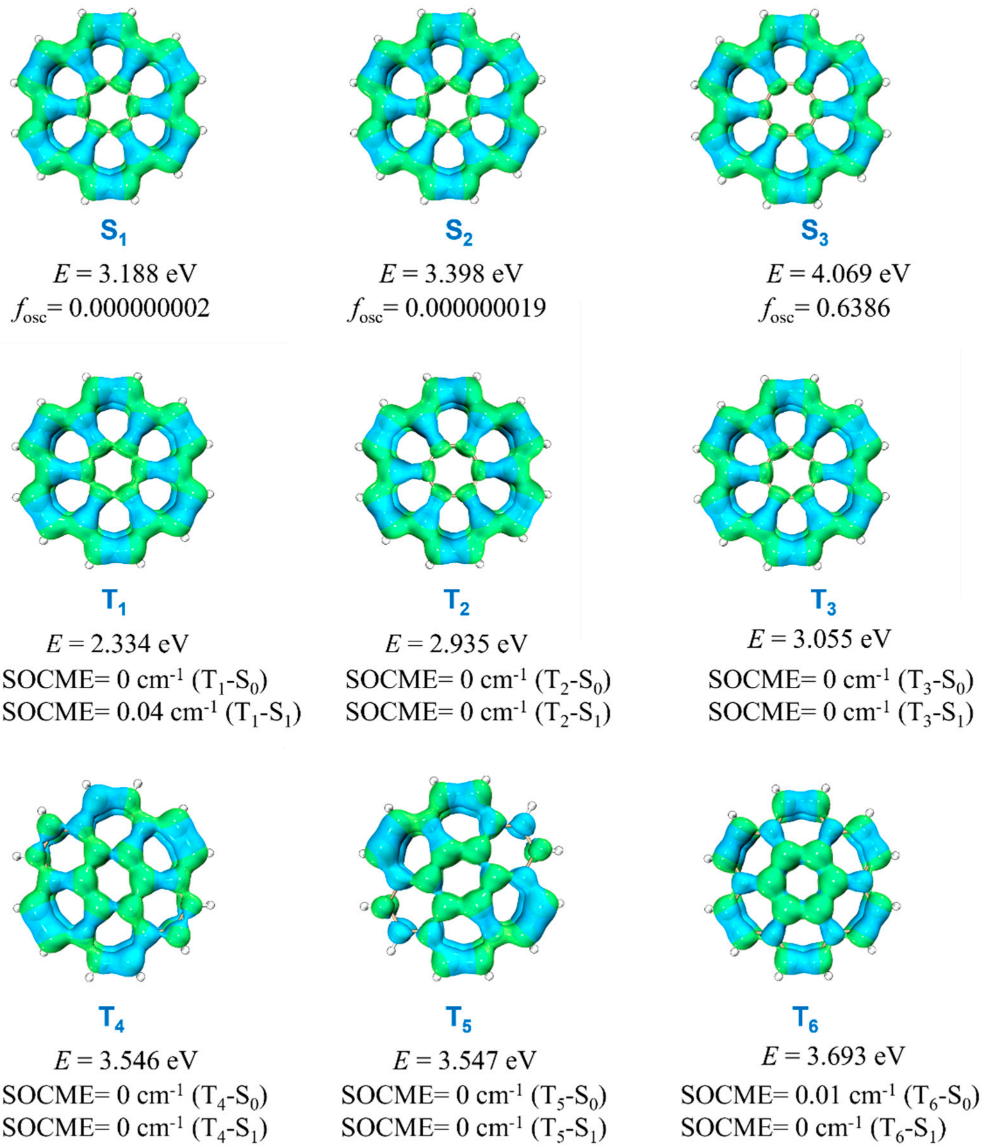

Figure S15. TD-DFT-calculated electron density difference of singlet and triplet excited states of Cor. The optimized geometry of Cor ground state was obtained by a DFT calculation on Gaussian 16 program (Revision A.03) using B3LYP functional and 6-31G(d,p) basis set. The singlet excited states and triplet excited states were calculated on ORCA 4.2.1 program with B3LYP/G functional and def2-TZVP(-f) basis set. Spin-orbit coupling (SOC) matrix elements between the singlet excited states and triplet excited states were calculated with spin-orbit mean-field (SOMF) methods on ORCA 4.2.1 program with B3LYP/G functional and def2-TZVP(-f) basis set.

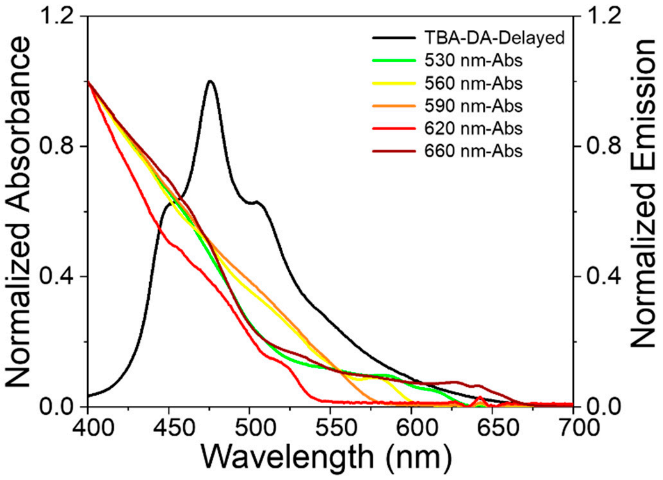

Figure S16. Room-temperature delayed emission (1 ms delay) spectra of TBA-DA-0.1% (black line) and absorption of several QDs of different emission wavelength.

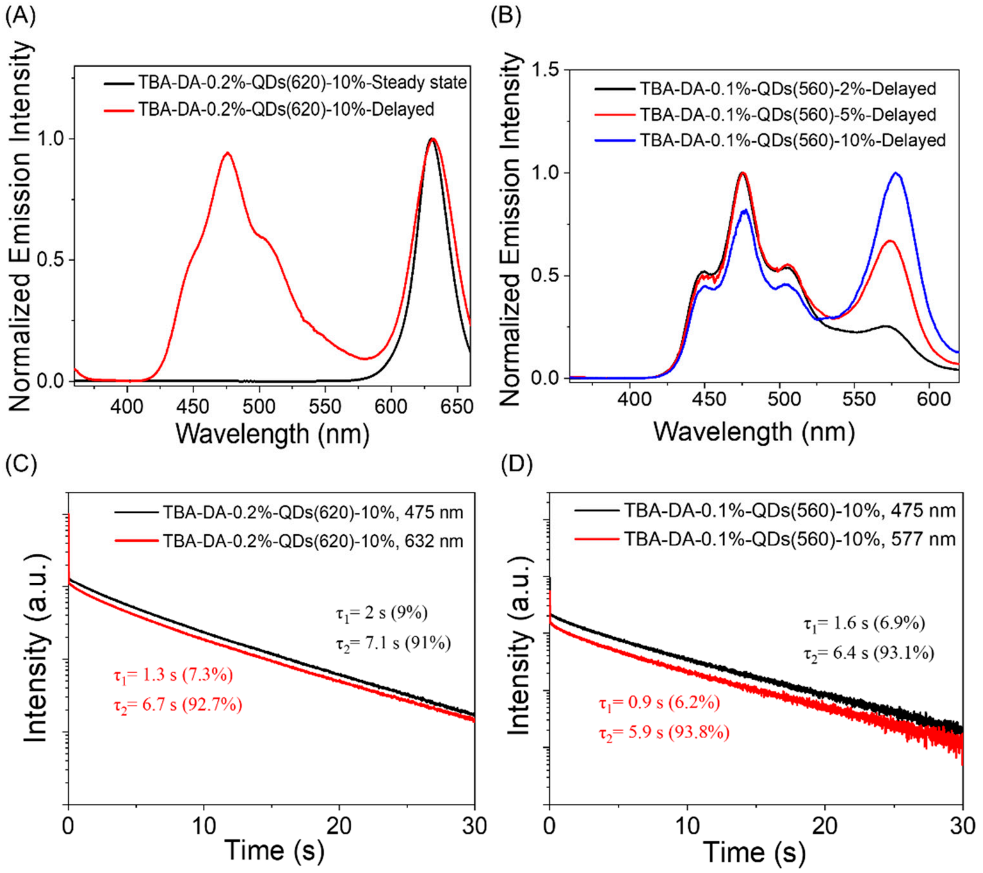

Figure S17. (A) Room-temperature steady-state (black line) and delayed emission (1 ms delay, red line) spectra and (C) emission decay monitored at 475 and 632 nm of TBA-DA-0.2%-QDs(560) three-component afterglow material. (B) Room-temperature steady-state (black line) and delayed emission (1 ms delay, red line) spectra and (D) emission decay monitored at 475 and 577 nm of TBA-DA-0.2%-QDs(560) three-component afterglow material.

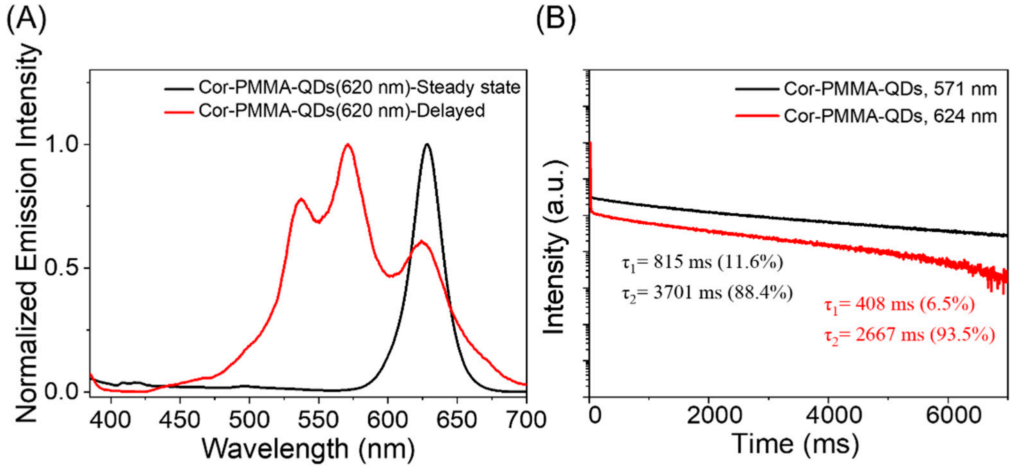

Figure S18. (A) Room-temperature steady-state and delayed emission (1 ms delay) spectra of Cor-PMMA-QDs(620) emulsion. (B) Room-temperature afterglow decay of Cor-PMMA-QDs(620) emulsion monitored at 571 nm and 624 nm.

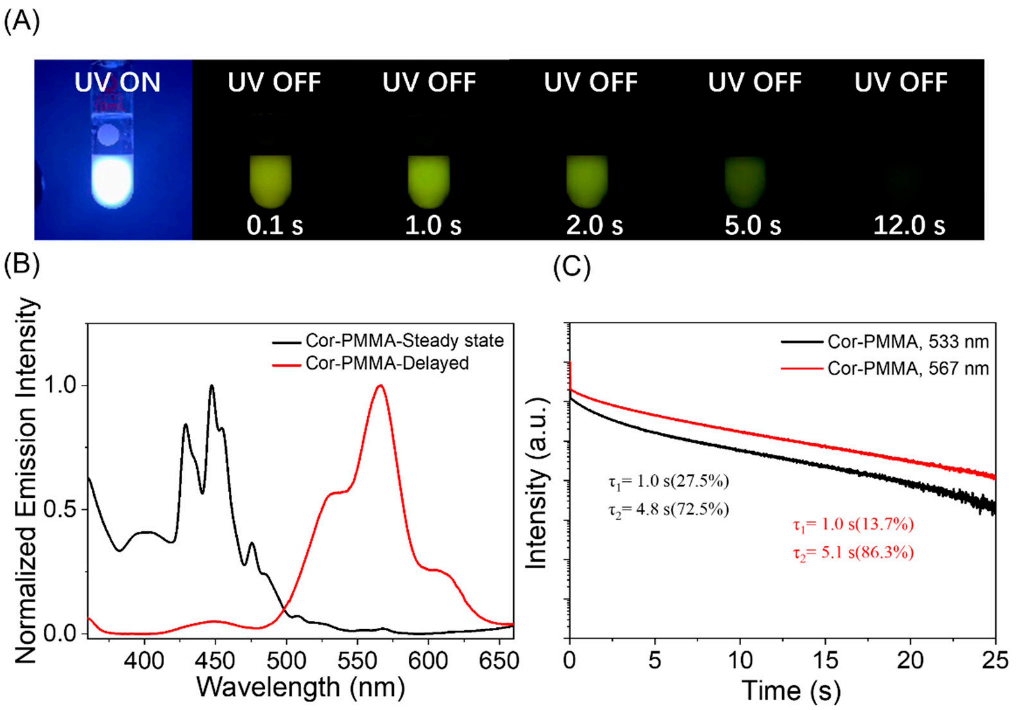

Figure S19. (A) Photographs of Cor-PMMA emulsion under 365 nm UV light and after turning the light off at room temperature. (B) Room-temperature steady-state and delayed emission (1 ms delay) spectra of Cor-PMMA emulsion. (C) Room-temperature afterglow decay of Cor-PMMA emulsion monitored at 533 nm and 567 nm.

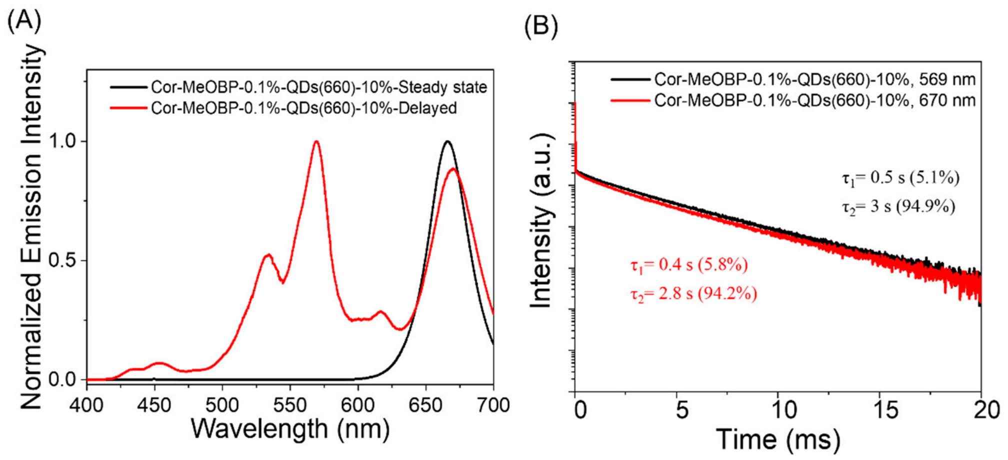

Figure S20. (A) Room-temperature steady-state (black line) and delayed emission (1 ms delay, red line) spectra and (B) emission decay monitored at 569 and 670 nm of Cor-MeOBP-0.1%-QDs(660) three-component afterglow material.
